# *Notes from the Field:* Identifying Risk Behaviors for Invasive Group A *Streptococcus* Infections Among Persons Who Inject Drugs and Persons Experiencing Homelessness — New Mexico, May 2018

**DOI:** 10.15585/mmwr.mm6808a5

**Published:** 2019-03-01

**Authors:** Sandra J. Valenciano, Chelsea McMullen, Salina Torres, Chad Smelser, Almea Matanock, Chris Van Beneden

**Affiliations:** ^1^Epidemic Intelligence Service, CDC; ^2^Division of Bacterial Diseases, National Center for Immunization and Respiratory Diseases, CDC; ^3^New Mexico Department of Health.

In the spring of 2018, the New Mexico Department of Health (NMDOH) contacted CDC about an increase in the number and prevalence of invasive group A *Streptococcus* (GAS) infections reported through New Mexico’s Active Bacterial Core surveillance (ABCs) system. From 2013 to 2017, the annual rate of invasive GAS infections increased approximately 120%, from 6.8 to 14.9 per 100,000 persons, approximately double the estimated national rate (*1*,*2*). In New Mexico, the prevalence of injection drug use (IDU) reported in the medical charts of patients with invasive GAS infection during this period (1,108 patients) increased approximately 200%, from 6.4% (nine of 141 invasive GAS infections) to 20.1% (62 of 308 invasive GAS infections), and the prevalence of reported homelessness among persons with invasive GAS infections increased 125%, from 3.6% (five of 141) to 8.1% (25 of 308). IDU is a known risk factor for GAS infections; however, specific behaviors causing the recent increase in the prevalence of IDU among patients with GAS infection are unknown. Although recent outbreaks of invasive GAS infection among persons experiencing homelessness have been reported in Canada, Europe, Arizona, and Alaska, homelessness is not a well-defined risk factor for invasive GAS infection in the United States; therefore, identifying specific behaviors that might increase the risk for infection in this group might help inform prevention efforts (*3*–*6*). NMDOH requested CDC assistance in characterizing GAS disease and specific high-risk behaviors among persons who inject drugs and persons experiencing homelessness to recommend potential public health interventions to reduce disease risk and transmission among these populations.

NMDOH and CDC received daily laboratory lists to identify patients hospitalized with GAS infection during May 1–23, 2018, at one of four hospital systems in Albuquerque and Santa Fe. A case was defined as illness with GAS cultured from any site (excluding throat and urine cultures) in an adult aged 18–65 years. Identified patients were interviewed using a standardized questionnaire focusing on known risk factors (e.g., exposure to ill children; crowding; IDU; and presence of underlying medical conditions, such as diabetes, chronic liver disease, and skin breakdown) and potential risk factors (e.g., poor hygiene, injection practices, and sharing of drug paraphernalia) for GAS infection and abstracted these data from their medical charts. The team interviewed personnel from organizations that care for persons who inject drugs and those experiencing homelessness to generate hypotheses for the increase in GAS infections among these groups.

Thirty-five patients with GAS infection were identified; 26 (74%) could be contacted and are included in the analysis. The mean patient age was 48 years (range = 24–63 years); 17 (65.4%) were male, seven (26.9%) were American Indian, and 15 (57.7%) identified as Hispanic/Latino (Table). Approximately half of the cases were identified as cellulitis, and approximately one third were identified as abscesses. Among known risk factors for GAS infections, skin breakdown, either recent (21; 80.8%) or current (20; 76.9%), was reported most frequently. Fifteen (57.7%) patients had been seen by a wound care provider in the month preceding their admission. Eight (30.8%) interviewed patients were experiencing homelessness, and three (11.5%) injected drugs. Persons experiencing homelessness did not report staying in crowded settings, such as shelters. However, recent or current skin breakdown was reported by six and five persons experiencing homelessness, respectively, and six reported having seen a wound care provider before their hospital admission. Reported injected drugs included heroin, cocaine, and methamphetamines, alone or in combination. All three persons who injected drugs reported injecting multiple times in the same day, two reported injecting multiple times with the same needle, and one reported sharing needles and licking the needle before injection.

**TABLE:** Characteristics of interviewed patients with group A streptococcal (GAS) infection (N = 26) — New Mexico, May 2018

Characteristic	No. (%)
**Sex**	
Male	17 (65.4)
Female	9 (34.6)
**Race**	
White	6 (23.1)
Black	1 (3.8)
American Indian	7 (26.9)
Asian/Pacific Islander	1 (3.8)
Multiracial	1 (3.8)
Unknown	10 (38.5)
**Ethnicity**	
Hispanic/Latino	15 (57.7)
Type of GAS infection*	
Cellulitis	15 (57.7)
Abscess	9 (34.6)
Osteomyelitis	4 (15.4)
Septic shock	3 (11.5)
Necrotizing fasciitis	2 (7.7)
Pneumonia	2 (7.7)
Bacteremia	1 (3.9)
Septic arthritis	1 (3.9)
**Risk factors for group A streptococcal infections**	
Skin breakdown in the last month	21 (80.8)
Current skin breakdown	20 (76.9)
Diabetes	9 (34.6)
Contact with ill children	7 (26.9)
Chronic hepatitis C	6 (23.1)
Cirrhosis of liver	6 (23.1)
Injection drug use	3 (11.5)
Contact with ill adults	2 (7.7)
Heart disease	2 (7.7)
Cancer	1 (3.9)
Chronic obstructive pulmonary disease	1 (3.9)
**Contact with health care system**	
Saw health care provider in the past year	22 (84.6)
Saw health care provider in week before illness	16 (61.5)
Saw wound care provider in last month	15 (57.7)

* Categories are not mutually exclusive.

Among 15 interviewed providers, barriers to good hygiene and appropriate skin care in persons experiencing homelessness and persons who inject drugs were noted, including limited access to clean running water, showers, or bathrooms; poverty; or alcohol and drug addiction. To prevent GAS infection, seven providers suggested developing educational tools for persons experiencing homelessness, persons who inject drugs, and personnel working with these populations.

The increased number of invasive GAS cases occurring among persons experiencing homelessness and those who inject drugs might have contributed to the overall increase in GAS in New Mexico from 2013 to 2017. However, the small number of persons interviewed for this study limit the ability to draw significant conclusions regarding specific risk behaviors that might increase the risk for GAS infection among these populations. Replicating this pilot investigation at other sites might help identify specific risk behaviors for acquiring GAS among these vulnerable populations. Because most patients had previous encounters with the health care system, it is important for providers who care for persons who inject drugs or are experiencing homelessness to be aware of the risk for severe GAS infections in these groups. In addition, educational material, that describes GAS symptoms, good hygiene and skin care, and safe injection practices, could benefit both patients and providers.
